# General practitioners’ self-reported competence in the management of sexual health issues – a web-based questionnaire study from Finland

**DOI:** 10.1080/02813432.2021.1934983

**Published:** 2021-07-13

**Authors:** Sanna-Mari Manninen, Katja Kero, Katariina Perkonoja, Tero Vahlberg, Päivi Polo-Kantola

**Affiliations:** a Department of Obstetrics and Gynecology, University of Turku, Turku, Finland; b Department of Health Promotion, Metropolia University of Applied Sciences, Helsinki, Finland; c Department of Obstetrics and Gynecology, Turku University Hospital and University of Turku, Turku, Finland; d Department of Clinical Medicine, Biostatistics, University of Turku, Turku, Finland

**Keywords:** Female, Finland, general practitioner, male, physician–patient relations, sexual health, surveys and questionnaires

## Abstract

*Objective* Although sexual problems are common, they are rarely brought up in appointments with general practitioners (GPs). We aimed to assess the barriers that hinder GPs from bringing up sexual health issues and to evaluate the need for education on sexual medicine. *Design* A web-based questionnaire was used. *Setting* Four fields were included: A) the self-reported competence in discussing sexual health and treating patients with these issues, B) the barriers to bringing up patients’ sexual health problems, C) the source of education on sexual medicine and D) the need for education on sexual medicine. *Subjects* A random sample of 1000 GPs in Finland (a response rate of 43.5%, *n* = 402). *Main outcome measures* GPs’ self-assessed competence in discussing and treating sexual health issues, related barriers to bringing up the topic and the reported need for education. *Results* The main reasons reported for not bringing up sexual health issues were shortness of the appointment time (85.6%), a lack of knowledge (83.6%) and a lack of experience with sexual medicine (81.8%). The male GPs reported better competence in discussing the issues and treating male patients, whereas the female GPs reported better competence in discussing the issues with female patients. No differences emerged between genders regarding treating female patients. Nearly 90% of the GPs expressed needing more education about sexual medicine. *Conclusions* Although the GPs reported good competence in discussing sexual health issues with their patients, several barriers to bringing up sexual health issues emerged. Continuing education was desired and could lessen these barriers.

**Key points**
- Only a few studies have evaluated the competence of general practitioners (GPs) in addressing sexual health issues with their patients.
- In our study, the GPs reported a high competence in discussing patients’ sexual health issues regardless of the patient’s gender.
- However, several barriers to bringing up sexual health issues in appointments emerged.
- A majority of the GPs expressed a need for continuing education about sexual medicine.

## Introduction

Sexuality is a central aspect in life [1]. According to a study with sexually active participants of all ages, over 50% of the men and over 40% of the women considered good sexual health to be highly significant for good quality of life [2]. Importantly, this result was also found among participants with moderate or poor health or with chronic illnesses [2]. However, due to the highly intimate and delicate nature of sexual problems, patients may have difficulty bringing them to attention in appointments [3,4]. Therefore, it is essential that health care providers feel confident about taking the initiative in addressing sexual health issues with their patients.

For many patients, general practitioners (GPs) are the key doctors to whom they want to present all their health problems. In addition, for some patients, GPs are the only accessible medical professionals. It has been reported that patients present an average of 2.6 (a range of 1–16) problems during one appointment [5], which requires GPs to be fluent in addressing patients’ problems. Approximately, 4.2% of these problems deal with sexual health issues [6]. However, many sexual problems are undertreated in primary health care [7], and it is not usually routine for GPs to address sexual health issues with their patients [8]. According to previous research, GPs very seldom take sexual histories [9,10]. Approaching and managing organic sexual health problems, such as sexually transmitted diseases, is also considered to be easier than addressing problems with sexual functions [11], presumably because it is often simpler to treat diseases and symptoms that have a more precise cure.

As sexual health issues are often complex and therefore time-consuming to address, a lack of time during an appointment is quite uniformly reported as one of the main barriers to doctors addressing patients’ sexual health issues regardless of the nationality or the specialty of the physicians included in both qualitative [8] and quantitative [9,12–14] studies. Furthermore, in studies entirely focused on GPs, a lack of knowledge about and experience with sexual medicine [9,11,13,15] have been reported as barriers. With one exception [11], these studies were carried out in Europe [9,13,15], but varied in study design and were relatively small in terms of the numbers of participants [11] or response rates [9]. In a pilot study conducted in the Lisbon region in Portugal, personal attitudes and beliefs as well as a lack of effective treatment were found to be important barriers [13]. In two small qualitative studies [8,11] and in a larger quantitative study [15], one major barrier was a lack of education in sexual medicine. Training in communication skills has also been found to promote discussing sexual health issues [16]. In some previous studies, the majority of GPs reported a need for continuing education in sexual medicine [12,13,17]. Neither medical degree education nor residency was considered to be a sufficient source of education [8,9,13,14,17,18]. Gender (where the patient represents the opposite gender) may produce a barrier; however, the findings in the literature are not unanimous [8,9,13,14,17,18]. Moreover, younger doctors seem to be more insecure in dealing with patients with sexual problems [14,19]. Of note is that cultural differences may also influence and form barriers.

The main objectives of our study were to assess GPs’ self-reported competence and the barriers to bringing up patients’ sexual health issues. In addition, we aimed to evaluate the need for education in this field.

## Materials and methods

A random sample of GPs who were registered as members of the Finnish Medical Association and who had reported working in a health center were enrolled in the present Sexual Medicine Education (SexMEdu) study. According to the Finnish Medical Association’s general policy, contact information was restricted to 1000 Finnish GPs. Of the cohort, 75 were excluded because they reported not being a part of the target group (e.g. were retired or belonged to another specialty). Of the 925 remaining GPs, 402 replied, resulting in a response rate of 43.5%. In terms of background information, gender (woman [*n* = 302, 75%]/man [*n* = 100, 25%]), age (27–39 years, *n* = 147/40–49 years, *n* = 111/50–65 years, *n* = 144 years), and the number of patients seen with sexual health issues per week (0, *n* = 77/1–5, *n* = 265/≥6, *n* = 60) were assessed. Replying to the questionnaire implied consent, and the Ethics Committee of Turku University approved the study protocol (44/2017).

Our study questionnaire included 21 questions adopted and slightly modified from the Portuguese SEXOS study by Alarcão et al. [13] with permission obtained from the Portuguese researchers. The modifications mainly consisted of changes to some response options or scales. The questionnaire was piloted with 11 physicians; their feedback was used to make amendments to the content. The questionnaire consisted of four independent fields (A–D, Appendix): A) the self-reported competence in discussing sexual health and treating patients with sexual health issues (seven separate questions), B) the barriers to bringing up sexual health problems during GPs’ appointments (10 separate questions), C) the source of education on sexual medicine (two separate questions) and D) the need for education on sexual medicine (two separate questions).

### Statistical analyses

Data is described using frequencies (percentages). A chi-square test was used to compare frequencies between the groups. In the analyses, each question in fields A and B was dichotomized (A: ‘poor’ or ‘quite poor’ *versus* ‘good’ or ‘quite good’; B: ‘much’ or ‘very much’ *versus* ‘some’ or ‘not at all’), except question number 7 in field A (‘How do you usually conduct sexual history taking?’), which was a multiple-choice question with several options (Appendix). Furthermore, the ‘cannot say’ responses in field B were omitted from the analyses. Field C question number 1 and D question number 2 were also multiple-choice questions with several options. Question number 2 in field C was dichotomized (‘insufficient’ or ‘quite insufficient’ *versus* ‘quite sufficient’ or ‘sufficient’). The associations between the GPs’ gender, age (27–39, 40–49 and 50–65 years), the number of weekly patients with sexual health issues (0, 1–5 and ≥6 patients), and the four fields of interests (A–D) were analyzed using multivariable logistic regression (each question was examined separately in each field in the analyses). The results are presented using adjusted odds ratios (ORs) with 95% confidence intervals (CIs). *p* Values less than .05 were considered statistically significant. Statistical analyses were performed using the SAS System for Windows version 9.4 (SAS Institute Inc., Cary, NC).

## Results

### A) The self-reported competence in discussing sexual health issues and treating patients

Overall, the GPs self-reported good competence in discussing sexual health issues with patients. If the patient addressed the issue, 96% of the GPs reported having no or only minor problems discussing the topic. Furthermore, the competence in discussing it with either male or female patients was similar (good or quite good: 71% *versus* 72%, respectively, *p* = .754). However, self-reported competence in treating male patients was evaluated more highly than that of treating female patients (65% *versus* 33%, respectively, *p* < .001).

Compared to the female GPs, the male GPs more often reported good or quite good competence in discussing sexual health and treating male patients’ sexual health issues. Similarly, the female GPs more often reported good or quite good competence in discussing these issues with female patients than the male GPs; however, there were no gender differences in terms of their self-reported competence in treating female patients. No differences emerged among the GPs’ different age groups. Furthermore, the more the GPs saw patients with sexual health issues weekly, the more competent they reported being in discussing and treating both male and female patients (Table 1). Only 37% of the GPs reported asking about patients’ satisfaction in sexual life, and no differences were found in terms of the GPs’ gender and age. The more the GPs saw patients with sexual health issues weekly, the more frequently they reported asking about satisfaction in sexual life (*p* < .001, 1–5 *versus* 0 OR 2.29, 95% CI 1.38–3.79, ≥6 *versus* 0 OR 3.93, 95% CI 2.00–7.73, ≥6 *versus* 1–5 OR 1.72, 95% CI 1.00–2.97). A majority (*n* = 349) of the GPs reported using open conversation as the method of taking a patient’s sexual history. Structured interviews were indicated 11 times, questionnaires 17 times and the option ‘I don’t take a sexual history’ 47 times.

**Table 1.** The competence in discussing sexual health issues and treating male and female patients’ sexual health issues (total *n* = 402).

	Discussing sexual health with male patients		Treating male patients’ sexual health issues		Discussing sexual health with female patients		Treating female patients’ sexual health issues	
	Poor or quite poor 28.9% (*n* = 116/402)		Poor or quite poor 34.8% (*n* = 140/402)		Poor or quite poor 27.9% (*n* = 112/402)		Poor or quite poor 67.2% (*n* = 270/402)	
Entire group	OR	95% CI	OR	95% CI	OR	95% CI	OR	95% CI
Gender	*p* < .0001		*p* < .0001		*p* < .001		*p* = .394	
women *versus* men	8.34	3.70–18.79	6.32	3.27–12.23	0.42	0.26–0.68	0.80	0.49–1.33
Age	*p* = .458		*p* = .373		*p* = .175		*p* = .479	
40–49 *versus* 27–39	1.28	0.72–2.25	1.47	0.85–2.55	1.57	0.88–2.80	0.72	0.43–1.22
50–65 *versus* 27–39	0.89	0.52–1.53	1.11	0.67–1.87	1.60	0.94–2.74	0.87	0.53–1.44
40–49 *versus* 50–65	1.44	0.81–2.56	1.32	0.76–2.29	0.98	0.56–1.71	0.83	0.49–1.41
Patients with sexual health issues weekly	*p* = .056		*p* = .002		*p* = .030		*p* = .057	
0 *versus* 1–5	1.57	0.88–2.80	1.83	1.05–3.20	1.46	0.85–2.52	1.32	0.74–2.35
0 *versus* ≥ 6	2.66	1.19–5.94	4.12	1.86–9.12	3.35	1.37–8.18	2.36	1.14–4.90
1–5 *versus* ≥ 6	1.70	0.86–3.35	2.25	1.15–4.41	2.29	1.02–5.13	1.79	1.00–3.19

In all questions the response was mandatory.

OR higher than 1 indicates worse self-reported competence (two categories: poor or quite poor *versus* good or quite good) in discussing sexual health or treating patients.

OR less than 1 indicates better self-reported competence in discussing sexual health or treating patients.

OR: odds ratio; multivariable logistic regression; CI: confidence interval

### B) The barriers to bringing up sexual health problems during GPs’ appointments

The four most important barriers to bringing up sexual health issues were shortness of the appointment time, lack of knowledge about sexual medicine, lack of experience with sexual medicine and sexual health issues not being a priority in the appointment. Compared to the male GPs, the female GPs were more likely to consider the lack of effective treatment and fear of failing to respond to patients’ sexual health issues to hinder bringing up the subject. In addition, personal attitudes and beliefs and lack of experience with sexual medicine showed tendencies. In contrast, among the male GPs, gender differences (where the patient represents the opposite gender) showed to be a higher barrier (Table 2). Only a few differences emerged between the GPs’ age groups (Table 2). The more the GPs saw patients with sexual health issues weekly, the fewer barriers hindered bringing up sexual health issues (Table 2).

**Table 2.** The barriers to bringing up sexual health issues during appointments (total *n* = 402).

	Shortness of the appointment time		Sexual health issues not being a priority in the appointment		Personal attitudes and beliefs		Personal discomfort when addressing sexual health issues		Lack of knowledge about sexual medicine		Lack of experience with sexual medicine		Lack of effective treatment		Fear of failing to respond to patients' sexual healthissues		Gender differences (where the patient represents the opposite gender)		Disability of the patient	
	Much or very much 85.6% (*n* = 344/397)		Much or very much 80.4% (*n* = 323/389)		Much or very much 17.2% (*n* = 69/387)		Much or very much 17.4% (*n* = 70/396)		Much or very much 83.6% (*n* = 336/391)		Much or very much 81.8% (*n* = 329/396)		Much or very much 54.7% (*n* = 220/374)		Much or very much 46.8% (*n* = 188/389)		Much or very much 21.1% (*n* = 85/383)		Much or very much 21.1% (*n* = 85/375)	
Entire group	OR	95%CI	OR	95%CI	OR	95%CI	OR	95%CI	OR	95%CI	OR	95%CI	OR	95%CI	OR	95%CI	OR	95%CI	OR	95%CI
Gender	*p* = .110		*p* = .091		*p*= .059		*p* = .150		*p* = .167		*p* = .068		*p* < .001		*p* < .001		*p* = .034		*p* = .161	
women *versus* men	1.68	0.89–3.19	1.67	0.92–3.01	1.96	0.97–3.95	1.61	0.84–3.06	1.60	0.82–3.09	1.76	0.96–3.23	2.32	1.43–3.76	2.36	1.45–3.85	0.56	0.33–0.96	1.55	0.84–2.86
Age	*p* = .139		*p* = .095		*p* = .044		*p* = .823		*p* = .986		*p* = .043		*p* = .428		*p* = .917		*p* = .457		*p* = .041	
40–49 *versus* 27–39	0.52	0.24–1.14	0.58	0.29–1.18	2.33	1.18–4.60	1.18	0.61–2.29	0.95	0.46–1.96	0.51	0.25–1.04	1.42	0.83–2.42	0.99	0.59–1.65	1.48	0.80–2.77	1.04	0.54–2.00
50–65 *versus* 27–39	0.50	0.24–1.03	0.49	0.25–0.94	1.87	0.96–3.63	0.97	0.52–1.80	1.02	0.50–2.05	0.43	0.22–0.85	1.08	0.66–1.77	1.09	0.67–1.76	1.26	0.70–2.26	1.93	1.09–3.43
40–49 *versus* 50–65	1.06	0.53–2.12	1.19	0.63–2.28	1.25	0.67–2.32	1.22	0.63–2.37	0.94	0.45–1.97	1.19	0.62–2.28	1.31	0.76–2.26	0.91	0.54–1.53	1.18	0.64–2.16	0.54	0.29–0.99
Patients with																				
sexual health issues weekly	*p* = .035		*p* = .375		*p* = 1.000		*p* = .018		*p* < .001		*p* < .001		*p* = .768		*p* = .026		*p* = .047		*p* = .520	
0 *versus* 1–5	0.41	0.21–0.80	1.18	0.57–2.45	1.01	0.51–2.02	1.56	0.84–2.87	2.01	0.75–5.36	3.01	1.14–7.96	0.86	0.49–1.52	1.21	0.71–2.07	1.20	0.66–2.19	0.80	0.40–1.58
0 *versus* ≥ 6	0.58	0.23–1.48	1.84	0.74–4.57	1.01	0.41–2.52	5.27	1.66–16.72	6.69	2.26–19.77	8.21	2.77–24.33	1.04	0.49–2.19	2.58	1.24–5.36	3.73	1.29–10.80	0.61	0.26–1.43
1–5 *versus* ≥ 6	1.41	0.60–3.34	1.55	0.76–3.19	1.00	0.47–2.12	3.39	1.16–9.89	3.33	1.68–6.63	2.73	1.40–5.31	1.20	0.66–2.20	2.13	1.16–3.91	3.10	1.17–8.18	0.76	0.39–1.48

The responses ‘cannot say’ omitted from the analyses.

OR higher than 1 indicates higher barriers (two categories: much or very much *versus* some or not at all) to bringing up sexual health issues during appointments.

OR less than 1 indicates lower barriers to bringing up sexual health issues during appointments.

OR: odds ratio; multivariable logistic regression; CI: confidence interval

### C) The source of education on sexual medicine

**Figure 1.**
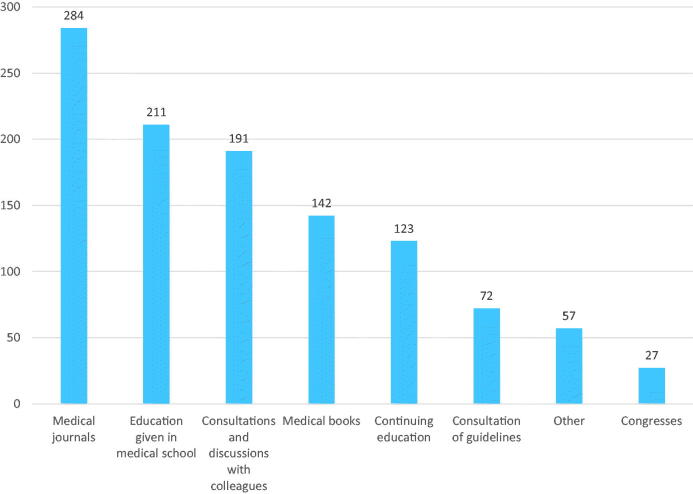
The general practitioners’ (total *n* = 402) self-reported sources of education on sexual medicine (more than one option could be chosen).

The reported sources of education on sexual medicine are illustrated in Figure 1. The most important source was medical journals (*n* = 284), followed by education given in medical school (*n* = 211) (Figure 1). Although medical school was reported as an important source of education, 82.6% of the participants found it insufficient (*n* = 187) or quite insufficient (*n* = 145). Compared to the male GPs, the female GPs more often reported that the education in medical school was insufficient (*p* < .001, OR 2.85, 95% CI 1.61–5.04). Furthermore, the GPs seeing 1–5 patients with sexual health issues weekly considered medical school to be insufficient as a source of education compared to those seeing ≥6 patients (OR 2.27, 95% CI 1.12–4.58, *p* = .023). No differences emerged between the various age groups (*p* = .205).

### D) The need for education on sexual medicine

**Figure 2.**
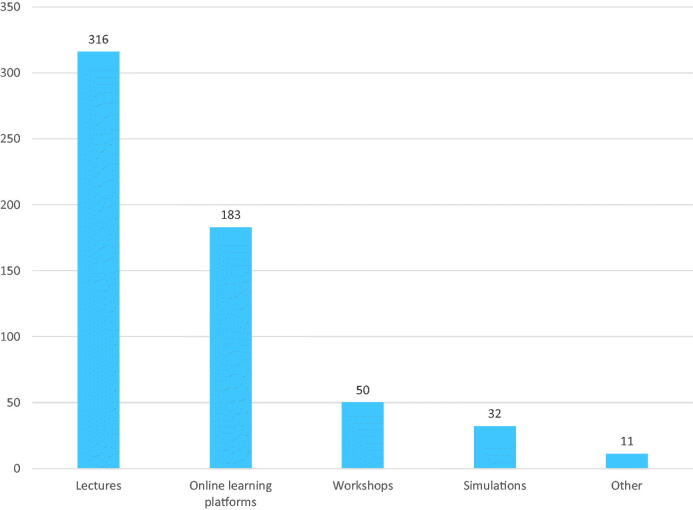
The general practitioners’ (total *n* = 402) self-reported preferences for the forms of continuing education on sexual medicine (more than one option could be chosen).

Overall, 87.8% of the GPs reported needing more education on sexual medicine. The female GPs were more likely to report a need for continuing education than the male GPs (*p* = .009, OR 2.34, 95% CI 1.24–4.42). In addition, the GPs seeing 1–5 patients with sexual issues weekly were more likely to report a need for education compared to the GPs seeing 0 patients with sexual issues (*p* = .023, OR 2.22, 95% CI 1.12–4.43). The responses to various forms of education are illustrated in Figure 2. The most preferred form of education was lectures (*n* = 316), followed by online learning platforms (*n* = 183) (Figure 2).

## Discussion

According to our study, the GPs reported good competence in discussing sexual health issues with their patients. However, treating sexual problems, especially those of female patients, was reported to be more difficult. Several factors hindered bringing up sexual health issues; most frequently, these factors were reported to be the shortness of the appointment time and a lack of knowledge about and experience with sexual medicine. Regarding the GPs’ gender, some differences emerged in bringing up patients’ sexual problems. Interestingly, the male GPs more often reported good competence in discussing sexual health and treating male patients than the female GPs; however, a similar gender advantage for female GPs was found only in discussing sexual health issues, not in treating them. There were no differences between the age groups in self-reported competence in discussing sexual health or treating patients. In addition, age had only marginal importance regarding the barriers to bringing up sexual health issues. The results were predictable concerning the numbers of patients with sexual health issues: the more the GPs saw patients with sexual health issues weekly, the better their self-reported competence was in discussing issues and treating these patients. Although the GPs used several sources of education, most of them considered their education on sexual medicine to be insufficient and reported a need for continuing education.

Our study expands on the current literature, as it was the first study of its kind conducted in Finland and, to the best of our knowledge, also in Scandinavia. Furthermore, our study is one of the few studies in the literature evaluating several aspects of the barriers to bringing up sexual health issues in GP appointments. One of the merits of the study is that it included a high number of participants. Our response rate of 43.5% was only moderate; however, it fell into the range of previous studies, from 16% [14] to 73.5% [13]. Thus, our data could be considered representative and comparable to other studies. We used a structured questionnaire and utilized a web-based program instead of a personal interview. Although this could result in hasty replies, the participants might be more honest in replies that are given anonymously. The web-based questionnaire was a practical tool to obtain responses from a large sample. In addition, it was programmed not to proceed if replies were missing, which ensured that the questionnaire was complete. Furthermore, we piloted our study questionnaire, which allowed us to amend the content. In our analyses, we also considered the effects of the GPs’ gender, age and number of weekly patients seen with sexual health issues. However, as we included only Finnish GPs, our results are not necessarily directly applicable to physicians in other countries and in other specialties. It is also plausible that GPs who are more involved with patients with sexual problems were more likely to have participated in the study. However, we assessed the numbers of patients with sexual health issues seen weekly, and GPs without these patients also participated in our survey. Therefore, it is unlikely that the GPs’ special interest in sexual medicine would have biased our results. As research into education about sexual medicine is limited and information is primarily only available from the past 15 years, we have mainly used articles from 2006 onward.

The literature is not consistent in terms of the preference for the same gender in consultations about sexual health issues [8,13,14,16,18,20]. In a UK study with 22 GPs [8], a US study with 98 GPs [18], and a Portuguese study with 50 GPs [13], a preference for the same gender was reported. We separately assessed the discussion and treatment of sexual health issues in female and male patients and confirmed the gender preference for GPs in discussing sexual health issues. However, concerning treatment, the same-gender preference was found only when treating male patients. One important reason could be that in our study, the treatment of female patients was generally considered more difficult than the treatment of male patients. Alarcão et al. [13] showed that 38.9% of male GPs and 56.7% of female GPs reported low confidence when managing sexual dysfunction in male patients. Concerning the management of female patients, the percentages were 44.4% and 37.9%, respectively. In a German study with 905 physicians working in urology and andrology, only a minority of GPs considered opposite-gender discordance; this was more often the case with female physicians (13.3%) than male physicians (7.5%) [14]. Furthermore, in an Italian study with 127 GPs addressing the management of erectile dysfunction, female GPs had a threefold higher probability of being uncomfortable when diagnosing erectile dysfunction [20]. In a multi-country European study with 366 participants, male trainees in various specialties felt more confident than female trainees in dealing with patients with sexual dysfunctions [17]. In addition, a recent Norwegian study with 152 GPs showed that male GPs were more reluctant to perform gynecological examinations on their patients compared to female GPs [21].

Previously, Schloegl et al. [14] and Ariffin et al. [19] showed that younger physicians were less confident in taking care of patients with sexual problems. However, in the above-mentioned study evaluating the management of erectile dysfunction, older GPs were found to be less likely to prescribe treatment [20]. In our study, the competence in discussing issues or treating patients was not dependent on age. Moreover, predictably, the competence in taking care of patients with sexual health issues was better among GPs who treated patients with these problems more frequently.

The most important barrier to bringing up sexual health issues in our study was shortness of the appointment time, which was consistent across gender and age groups. This finding confirmed that dealing with sexual health issues is time-consuming, in concordance with the Alarcão et al. study [13]. Additionally, in a study by Schloegl et al. [14], 61% of the participants mentioned a lack of time as a barrier to addressing patients’ sexual health issues, more so among female doctors compared to male doctors and among young doctors compared to older ones. Time constraints were also identified as key barriers in the Gott et al. [8] study with 22 GPs and in the Byrne et al. [9] study with 61 GPs. In addition, a frequent finding in previous studies that the sexual health issue was not considered to be the priority of the appointment was also found in our study. This further illustrates the importance of allocating sufficient time for patients.

A lack of knowledge about and lack of experience with sexual medicine were also important barriers to bringing up sexual health issues. This was especially stated by those GPs who had fewer patients reporting sexual issues and therefore less experience treating them. Similar results have been reported previously. In the Byrne et al. [9] study, 31% of the participants cited a lack of knowledge and 62% a lack of training as important barriers. In other studies, a lack of knowledge [13], a lack of experience [11,13] and a lack of training [13,15] were rated as relevant barriers. In a recent Austrian study with 391 medical students, 96.9% of the respondents reported not being instructed in sexual history taking [22].

We found that personal attitudes and beliefs, as well as discomfort when addressing sexual health issues, were only minor barriers to bringing up sexual health issues. Respectively, older GPs and GPs with lower numbers of patients with sexual health issues were more likely to report encountering these barriers. This finding was in line with the results presented by Byrne et al. [9] and Schloegl et al. [14]. In contrast, in the Alarcão et al. [13] study, personal attitudes and beliefs showed to be major barriers, whereas discomfort when addressing sexual health issues was not important [13]. Furthermore, lack of effective treatment has been reported as one of the main difficulties when treating sexual problems, especially for female patients [12]. In our study, particularly according to the reports of the female GPs, the lack of effective treatment and fear of failing to respond to patients’ sexual health issues showed to be barriers. The latter is a novel finding. One explanation for this finding could be that based on clinical experience, female GPs more often treat women with sexual health issues compared to male GPs and there are fewer available treatments for women than for men.

Although the GPs in our study referred to several educational resources for sexual medicine, they mainly used medical journals, medical school and consultations with colleagues. In concordance with a Swiss [23] and a Portuguese [13] study, we also found a great need for sexual education: almost nine out of 10 GPs expressed a need for continuing education. As for educational sources, previous literature has suggested didactic teaching and lectures, workshops, panel discussions and roleplay with standardized patients being practical and effective educational tools [24]. The GPs in our study also reported using some of these tools; however, lectures or online learning platforms were preferred over other alternatives.

According to our study, GPs reported a high competence in discussing sexual health issues with their patients regardless of the patient’s gender. However, self-reported competence in treating female sexual health issues in particular was lower. Furthermore, several barriers to bringing up sexual health issues emerged. Our study clearly showed a great need for continuing education in sexual medicine, as most of the GPs considered their education to be insufficient and expressed a need for continuing education.

## Appendix STUDY QUESTIONNAIRE

*A) The self-reported competence in discussing sexual health and treating patients with sexual health issues (seven questions)*

**1. How easy is it for you to discuss sexual health issues if your patient addresses the subject?**

[ ] Not a problem

[ ] A minor problem

[ ] A moderate problem

[ ] A major problem

[ ] Cannot say

**2. How do you classify your competence in discussing sexual problems with male patients?**

[ ] Good

[ ] Quite good

[ ] Quite poor

[ ] Poor

**3. How do you classify your competence in discussing sexual problems with female patients?**

[ ] Good

[ ] Moderate

[ ] Quite poor

[ ] Poor

**4. How do you classify your competence in treating male patients’ sexual problems?**

[ ] Good

[ ] Quite good

[ ] Quite poor

[ ] Poor

**5. How do you classify your competence in treating female patients’ sexual problems?**

[ ] Good

[ ] Moderate

[ ] Quite poor

[ ] Poor

**6. When you take a sexual history, do you explore how satisfied the patient is with his/her sexual life?**

[ ] Always

[ ] Usually

[ ] Seldom

[ ] Never

**7. How do you usually conduct sexual history taking** (*you can choose more than one option)***?**

[ ] Open conversation

[ ] Questionnaire

[ ] Structured interview

[ ] I don´t take a sexual history

*B) The barriers to bringing up sexual health problems during GPs´ appointments (ten questions)*

**Bringing up sexual health issues with patients hinders:**

*C) The source of education on sexual medicine (two questions)*

**1. From which sources have you gained your knowledge about sexual medicine that you use in your patient work? *(you can choose more than one option)***

[ ] Medical books

[ ] Medical journals

[ ] Continuing education

[ ] Congresses

[ ] Consultation of guidelines

[ ] Education given in medical school

[ ] Consultations and discussions with colleagues

[ ] Other: _________________________________________________________________________

**2. How sufficient do you classify medical school as a source of education when considering your sexual medicine competence?**

*D) The need for education on sexual medicine (two questions)*

**1. Do you feel a need for continuing education on sexual medicine?**

[ ] Yes

[ ] No

**2. If you answered yes, in which form would you prefer receiving continuing education (you may choose more than one option)?**

[ ] Lectures

[ ] Workshops

[ ] Simulations

[ ] Online learning platforms

[ ] Something else, what?
